# Early Microbes Modify Immune System Development and Metabolic Homeostasis—The “Restaurant” Hypothesis Revisited

**DOI:** 10.3389/fendo.2017.00349

**Published:** 2017-12-13

**Authors:** Michael J. Nash, Daniel N. Frank, Jacob E. Friedman

**Affiliations:** ^1^Department of Pediatrics, Section of Neonatology, University of Colorado Anschutz Medical Campus, Aurora, CO, United States; ^2^Division of Infectious Diseases, Department of Medicine, University of Colorado Anschutz Medical Campus, Aurora, CO, United States; ^3^Department of Biochemistry and Molecular Genetics, University of Colorado Anschutz Medical Campus, Aurora, CO, United States

**Keywords:** pregnancy, innate immunity, microbiome, non-alcoholic fatty liver disease, proteobacteria

## Abstract

The developing infant gut microbiome affects metabolism, maturation of the gastrointestinal tract, immune system function, and brain development. Initial seeding of the neonatal microbiota occurs through maternal and environmental contact. Maternal diet, antibiotic use, and cesarean section alter the offspring microbiota composition, at least temporarily. Nutrients are thought to regulate initial perinatal microbial colonization, a paradigm known as the “Restaurant” hypothesis. This hypothesis proposes that early nutritional stresses alter both the initial colonizing bacteria and the development of signaling pathways controlled by microbial mediators. These stresses fine-tune the immune system and metabolic homeostasis in early life, potentially setting the stage for long-term metabolic and immune health. Dysbiosis, an imbalance or a maladaptation in the microbiota, can be caused by several factors including dietary alterations and antibiotics. Dysbiosis can alter biological processes in the gut and in tissues and organs throughout the body. Misregulated development and activity of both the innate and adaptive immune systems, driven by early dysbiosis, could have long-lasting pathologic consequences such as increased autoimmunity, increased adiposity, and non-alcoholic fatty liver disease (NAFLD). This review will focus on factors during pregnancy and the neonatal period that impact a neonate’s gut microbiome, as well as the mechanisms and possible links from early infancy that can drive increased risk for diseases including obesity and NAFLD. The complex pathways that connect diet, the microbiota, immune system development, and metabolism, particularly in early life, present exciting new frontiers for biomedical research.

## Introduction

**Figure 1:**
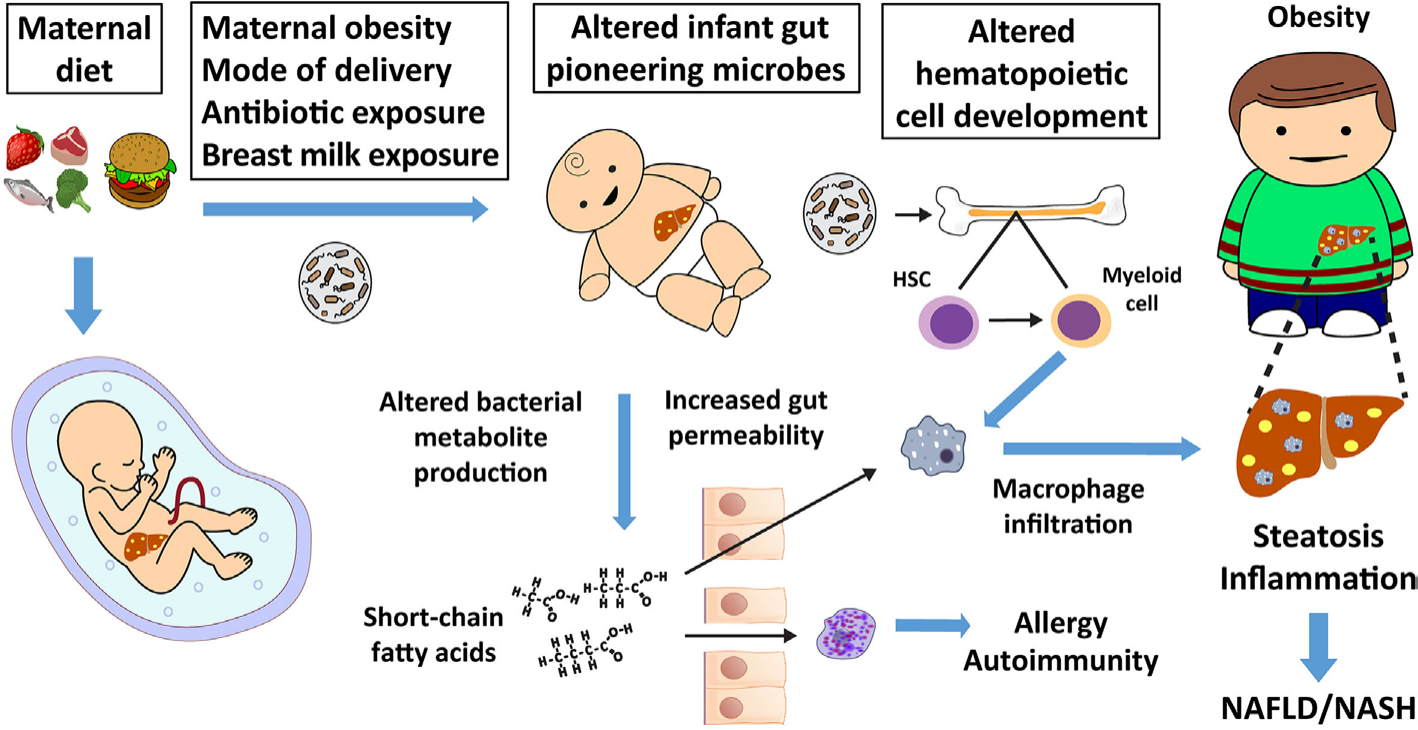
Maternal nutrition affects the fetal liver, microbiome, and offspring immunity, thereby increasing non-alcoholic fatty liver disease (NAFLD) and obesity risk. Maternal diet, mode of delivery, early offspring diet, and antibiotic exposure alter the infant’s pioneering microbiota and metabolite production. Changes in short-chain fatty acid production by the microbiota influence gut permeability, which can allow short-chain fatty acids and bacterial metabolites to directly influence adaptive and innate immune cell function and development. Altered adaptive immunity can lead to atopy, allergy, and autoimmunity. Microbes or their products alter hematopoietic stem cell (HSC) differentiation to macrophage progenitor cells in bone marrow and other tissues (17), which influences innate immunity. Altered innate immunity predisposes offspring to dysbiosis, macrophage activation, inappropriate macrophage infiltration, childhood obesity, and NAFLD, which often presents as non-alcoholic steatohepatitis (NASH) in obese youth.

Microbes introduced through the mother can serve as regulators of the infant immune system by contributing to and altering the composition and diversity of the infant gut microbiota. Because neonates have a limited capacity to initiate an immune response, the blooming of “pioneering” microbes in the neonate exerts critically important effects on postnatal immune responses that can be more persistent than those resulting from microbiota disruption during adult life, highlighting the neonatal period as a critical developmental window. While many factors alter the microbiome throughout life, the early infant pattern of microbiome development can have life-long implications for disease risk (1, 2) and the timing and dynamics of bacterial colonization later in development. In the first 1,000 days of life, many biological systems are established and are developmentally driven in part by environmental stimuli. The developmental origins of health and disease paradigm suggests that nutrient deficiency as well as nutrient excess *in utero* and in early infancy increases susceptibility to metabolic disease later in life (3, 4) and across generations (5). Maternal obesity and poor diet can influence the types and abundance of pioneering microbes. Changes in the early microbiome are associated with inflammatory diseases, such as asthma, allergies, and increased obesity risk (1, 6, 7). Early microbes affect the liver and other organs through direct communication *via* the portal system, through alterations in metabolite production or gut barrier integrity, and the hematopoietic immune cell axis (Figure 1). In rodents, non-human primates, and in human infants born to obese mothers or those consuming a high-fat diet (HFD), infant dysbiosis increases risk for both obesity and non-alcoholic fatty liver disease (NAFLD), a prevalent and harmful disease in children and adults with limited treatment options. Improper “training” of the innate immune system by microbial crosstalk with the immune system has been implicated in obesity and NAFLD (8, 9). Given that inoculation of obese human or obese mouse microbiota to germ-free (GF) mice alters the assembly of their intestinal microbiome in a manner that favors adipogenesis and inflammation (10–16), modifications to the structure of the infant intestinal microbiome by maternal obesity is plausible and might carry similar metabolic risk to the infant.

## Origins of the Gut Microbiota

Current data suggest that the microbiome might begin to assemble earlier in development than previously thought. Some have even suggested that the infant gut microbiome is partially established during pregnancy. Supporting this idea are reports that bacterial DNA is detectable in placental tissue (18) and amniotic fluid (19). Additionally, microbes have been detected in infant meconium (20, 21) wherein the bacterial composition of meconium is about 61% shared with the bacterial composition of amniotic fluid, further suggesting bacterial colonization *in utero* (20). These findings challenge the idea that the infant develops in a sterile environment. However, functional studies of the microbiota inhabiting these tissues are lacking, as many studies have only documented the presence of bacterial DNA, rather than viable bacteria, in embryonic tissues (22). Whether bacterial DNA detected during an uneventful, healthy pregnancy represents a transient exposure to microbes or microbial products (akin to transient bacteremia occurring in healthy adults), a potentially pathogenic phenomenon, or nascent assembly and succession of a microbial community is unknown.

## Effect of Diet on the Microbiota—The “Restaurant” Hypothesis

The concept that the first nutrient availability dictates the community structure of the intestine suggested a hypothesis, first termed the “Restaurant” hypothesis (23, 24), which proposes that nutritional stress can alter the original colonizing bacteria, particularly *Escherichia coli*, and signaling pathways controlled by microbial mediators. Since bacterial species grow at different rates under nutrient-limiting conditions, the rate of growth of each species during colonization can be modulated by competitive metabolic interactions within the intestine (25). *E. coli* normally represents only a small fraction of the human adult gut ecosystem; however, in the neonatal gut, its prevalence is higher (26). Under healthy conditions, vaginally delivered infants born to normal-weight mothers are initially colonized by mostly facultative anaerobic bacteria including species of *Escherichia, Staphylococcus*, and *Streptococcus* (21). *E. coli* is metabolically flexible and has a short doubling time, making this bacterium highly adaptable and allowing it to flourish in the presence of oxygen and nitrogen. Due to these attributes, its presence might explain significant functional variability in the human gut microbiome (27). Presumably, by consuming oxygen, altering the pH, lowering the redox potential, and producing carbon dioxide and nutrients, *E. coli* make the habitat in the gut suitable for colonization by strict anaerobes.

Although certain early-colonizing gut bacteria are thought to have evolved mechanisms that influence the growth of other microbes and maturation of the host immune system, nutrients can influence the gut microbial community by regulating the host gut epithelium (28). This creates specific niches for the early gut microbiota and for other microorganisms with similar nutrient biochemical capacity. A recent study reported that the microbiota is fundamentally shaped by the availability of nitrogen and microbial strategies for obtaining nitrogen in the gut (29), suggesting that the competitive advantage of certain bacteria in obtaining nitrogen can shape microbiota composition. This result reinforces the restaurant concept; what feeds the developing microbiota determines its composition, and hence its function.

Evidence that maternal diet is a significant driver of the infant microbiota composition comes from studies in non-human primates demonstrating that maternal HFD, independent of body habitus, results in the loss of key bacteria in offspring including decreased *Campylobacter, Helicobacter*, and *Bacteroidetes*, as well as decreased overall bacterial diversity when compared with control diet-fed mothers (2). This dysbiosis was not fully corrected by weaning offspring onto a control diet at a time when the microbiome is thought to be stabilized (2). Evidence based on diet recall suggests that HFD in human mothers affects the neonatal microbiota (30); while in a different study (31), feeding pregnant mice an HFD altered microbiota composition in the offspring, including an increase in Lachnospiraceae and Clostridiales, despite offspring being weaned onto a control diet. In addition to exhibiting dysbiosis, these offspring also had worse outcomes in models of infection, autoimmunity, and allergic sensitization. This has important clinical relevance as the early-colonizing bacteria, driven by maternal diet, can have a long-lasting effect on the commensal microbe population in offspring, setting the stage for increased risk of immunologic and metabolic disease. It is also important to note that maternal diet impacts multiple metabolic systems that can contribute to obesity, such as behavior and appetite (32).

With regard to specific gut bacteria in infants born to obese mothers, we reported that vaginally delivered, exclusively breastfed infants with no neonatal or postnatal exposure to antibiotics born to mothers with obesity had a significant 50% reduction in Gammaproteobacteria at 2 weeks of age compared with infants born to normal-weight mothers (33). This relative depletion in Proteobacteria was also reported in 2-day-old infants delivered vaginally, but not by cesarean section, to overweight/obese mothers (34). These results suggest that differences in the relative abundance of Proteobacteria might stem from vertical transmission of microbiota from the mother, but this requires confirmation of the mothers’ bacteria. Additional studies (35, 36) have suggested that maternal obesity can alter early gut microbes in the offspring, although factors such as antibiotic use, cesarean section delivery, and weight gain during pregnancy might confound maternal obesity as a factor impacting early offspring microbiota composition. Human milk oligosaccharides (37) and secretory IgA production (38) are involved in the selective suppression of Proteobacteria during establishment of the early gut microbiota. A delayed or reduced Proteobacteria content might have important consequences on lipopolysaccharide (LPS) production that is critical for immune education as discussed below.

## Changes in Early Postnatal Microbiota can Alter Disease Risk

Maternal obesity is frequently associated with cesarean delivery, and this mode of delivery carries increased risk of pediatric obesity (39). In addition, early antibiotic exposure and neonatal/infant diet influence the initial gut microbial community, and these changes are at least somewhat predictive of higher risks of allergy, atopy, and obesity (28, 29, 40, 41). Breastfeeding alters the composition of *Bifidobacteriaceae* and *Lactobacillaceae* in the early infant gut and promotes growth of important pro-inflammatory bacteria when compared with formula feeding (42, 43). Besides being a potential source of bacteria, human milk contains human milk oligosaccharides, a group of unconjugated glycans resistant to human enzymatic digestion which act as prebiotics for certain infant gut bacteria such as *Bifidobacterium*. Microbiota in breast milk from mothers with obesity have been shown to have less community diversity, with increased *Staphylococcus* and decreased *Bifidobacterium*, than that of normal-weight mothers (44). However, whether this is a true microbial community with a low-biomass relative to the gut, or whether these findings represent contamination by maternal skin or other body sites needs clarification. An urgent question that remains unanswered is whether maternal phenotype (i.e., maternal obesity or diabetes, or maternal diet) can also modify disease risk by altering the glycobiome of human milk.

## Role of the Microbiota in Innate Immunity

How the early microbiome impacts the immune system in the short- and long-term remains a critical concern. The microbiome has been implicated in altering macrophage development and polarization and an extensive area of research focuses on how bacterial metabolites, such as short-chain fatty acids, regulate macrophage function (45). A recent study showed that monocytes derived from newborn infants born to obese mothers displayed reduced LPS responsiveness, associated with altered cytokine promoter methylation (46), suggestive of early programming of the innate immune system by maternal obesity. An important role for early nutrition in the control of immune function is the fermentation of dietary polysaccharides to short-chain fatty acids by the microbiota. Acetate, proprionate, and butyrate have been shown to remodel regulatory T cell expansion in mice by inhibition of histone deacetylase activity in the FoxP3 promoter (47), as well as by causing histone deacetylase inhibition in murine macrophages that dampens immunity (48), and enhancing chemotaxis in neutrophils from rats (49), thereby skewing inflammation. In a murine model, oral administration of acetate during pregnancy was sufficient for the priming of FoxP3^+^ regulatory T cells and preventing allergic airway inflammation in the adult offspring (50), suggesting that *in utero* exposure to maternal gut microbial metabolites contributes to the development of immune function in the offspring. Although the mechanisms are poorly understood, the maternal microbiota has the ability to directly induce and/or regulate the offspring immune system (51). Using a reversible colonization model, microbial constituents, such as aryl hydrocarbon ligands, were shown to induce major transcriptional changes in the fetal gut and enhance the cellularity of the innate immune system (52). Fetal murine intestinal macrophages are constitutively non-responsive to LPS (53), whereas shortly after birth, acquisition of LPS resistance coincides with spontaneous activation of intestinal epithelial cells. This activation occurred in mice born vaginally, but not in offspring delivered by cesarean section or in toll-like receptor 4-deficient mice. These findings, coupled with local epithelial endotoxin measurements, identified LPS as a stimulatory agent (53) and suggest that epithelial activation shortly after birth is critical to maintain intestinal homeostasis.

The significant increase in common myeloid progenitor cells derived from bone marrow in diet-induced obese mice is associated with an altered microbiota composition (45). A key study has shown that the addition of microbes to gestating GF mice increases the prevalence of certain groups of intestinal innate immune system cells in offspring and also alters these cells’ transcriptional output (54), suggesting that maternal microbes alter function of the offspring immune system. A recent study has also shown that GF mice have compromised hematopoiesis and a decrease in several lines of myeloid cells in bone marrow and other tissues (17). The decrease in monocyte and granulocyte cells in bone marrow was reversible upon restoring the bacterial community *via* gavage in conjunction with heat killed *E. coli*, but not in conjunction with short-chain fatty acids (17). In a murine model, maternal HFD has also been shown to restrict the expansion and renewal of fetal hematopoietic stem cells by altering the transcriptional output of genes regulating metabolism, stress response, proliferation, and other functions (55). Importantly, these findings suggest that early microbes or maternal diet might alter immune function through alterations in hematopoietic cell development.

## Early Gut Dysbiosis is Associated with Elevated Risk for NAFLD

Gut microbes and inflammation play an active role in promoting the progression, and possibly the initiation, of NAFLD in children and adults (56–58). NAFLD describes a spectrum of liver abnormalities, ranging from simple hepatic steatosis to the more severe non-alcoholic steatohepatitis (NASH), with varying degrees of inflammation and fibrosis that can lead to cirrhosis. NAFLD affects 40% of all adults in the USA and well over 80% of adults with obesity (59). Importantly, NAFLD also affects approximately 34% of obese children ages 3–18 in North America and half have already progressed to NASH at the time of diagnosis (60–62). In addition, NAFLD is one of the fastest growing causes for liver cancer in the USA (63).

Intestinal dysbiosis has been correlated with NAFLD in children and adults; however, whether early life microbial composition influences hepatic fat accumulation and immunity before the disease occurs in humans is unclear (61, 64, 65). Our human studies (66), and those of others (67), using MRI/MRS, have documented that maternal BMI predicts newborn intrahepatocellular lipid storage, suggesting risk factors for pediatric obesity/NAFLD begin in early life (even *in utero*) and might permanently change the body’s structure, physiology, immune system, and metabolism, leading to an increased lifetime disease risk (68). A murine study showed that maternal HFD promoted increased liver adiposity, increased pro-fibrinogenic gene expression, and epigenetic changes in many genes in their offspring compared with offspring born from control diet-fed dams (69). This study also found that maternal HFD changed the microbial profile in offspring, significantly lowering the microbial biodiversity within a sample (69).

Highlighting the role of microbiota in the development of NAFLD is a recent study in mice showing that HFD-induced NASH can be attenuated by fecal transplant from chow-fed donors, as evidenced by a decrease in liver adiposity, and decreased inflammatory cytokines (70). Many other studies in rodent models similarly showed that probiotic treatment can slow NAFLD progression and help reverse the disease (71, 72). An increase in blood microbial DNA and a decrease in microbial biodiversity in patients with obesity was associated with liver fibrosis compared with patients without fibrosis (73). Therefore, there seems to be a distinct microbial community indicative of NAFLD rather than solely of obesity, which might suggest more specific microbial mechanisms that promote NASH.

Supporting the idea that prebiotics can be therapeutic is a study showing that bovine milk oligosaccharides prevented HFD-associated increases in gut permeability, dysbiosis, and obesity in mice (74). Furthermore, addition of the oligosaccharides decreased macrophage infiltration into adipose tissue, even in the setting of an HFD (74). A recent murine study reported that reversal of dysbiosis induced by maternal HFD and resulting pathologies might be possible by bacterial supplementation of defined microbial species in the offspring early in development, but not later in life (75). The study demonstrated not only that a maternal HFD causes dysbiosis, social problems, and defects in synaptic plasticity in offspring, but also that these defects can be reversed by administering *Lactobacillus reuteri* to the dysbiotic offspring who lacked this bacterium during weaning (75). This suggests that if definitive effects of the loss or the presence of certain bacteria can be identified, the negative effects of dysbiosis might be correctable with the relatively simple treatment of a probiotic in the early stages of life. While evidence suggests that microbe composition is alterable later in life by changes in the diet (76), the microbiota have different impacts on physiology during adulthood that make changes in composition less functionally relevant than changes in early life.

## Summary

Despite considerable intra- and inter-personal variations in the infant microbiota, keystone species in the neonatal microbial community can be patterned by diet and can have life-long effects on immunity and disease pathways. Specifically, early life bacterial species composition might be driven by nutrient availability, which supports the “Restaurant” hypothesis. This concept is clinically relevant because bacteria and their metabolites seem to influence development of the immune system, which impacts many aspects of physiology. Microbial-derived factors shaping macrophage metabolism, transcription, and polarization toward functional phenotypes are needed in the gut and other tissues to ensure homeostasis. Remarkably, animal models fed an HFD during pregnancy are programmed for inflammation, even if the offspring are switched to a normal diet at weaning. Legacy effects of maternal obesity or diet exposure might direct the development of the infant microbiota and innate immunity, and underlie common disorders including obesity and NAFLD. Delineating the links between microbiota function and composition in early life will help draw mechanistic connections between altered microbiota composition and disease risk. Thus, advancing our understanding of early life contributors to the microbiota and metabolic and inflammatory diseases is a critical and unmet need, and should motivate future studies.

## Author Contributions

Both MN and JF participated in drafting, writing, and editing the manuscript. DF participated in writing and editing the manuscript.

## Conflict of Interest Statement

JF is a consultant and recipient of a grant from Janssen Pharmaceuticals. MN and DF declared no conflict of interests.
